# Supplementary material for: Small molecule inhibitors of the Dishevelled‐CXXC5 interaction are new drug candidates for bone anabolic osteoporosis therapy
Source: EMBO Mol Med. 2016 Mar 3;8(4):375–87. doi: 10.15252/emmm.201505714 (PMC4818757; doi:10.15252/emmm.201505714)
Supplement: Supplementary file 1 — Appendix [file EMMM-8-375-s001.pdf]

APPENDIX TABLE OF CONTENTS

**SUPPLEMENTARY METHODS**	**Page 2**
**APPENDIX SCHEME S1**	**Page 3**
**APPENDIX TABLE S1**	**Page 8**
**APPENDIX TABLE S2**	**Page 8**
**APPENDIX TABLE S3**	**Page 10**
**APPENDIX TABLE S4**	**Page 11**
**APPENDIX TABLE S5**	**Page 11**
**APPENDIX FIGURE S1**	**Page 12**
**APPENDIX FIGURE S2**	**Page 13**
**APPENDIX FIGURE S3**	**Page 14**
**APPENDIX FIGURE S4**	**Page 16**
**APPENDIX FIGURE S5**	**Page 17**
**APPENDIX FIGURE S6**	**Page 19**
**APPENDIX REFERENCES**	**Page 21**

APPENDIX

SUPPLEMENTARY METHODS

Dvl PDZ domain purification

E. coli strain BL21 (DE3) was transformed with plasmid DNA encoding Dvl-1$^{247\text{-}341}$ (the Dvl PDZ domain), and the cells were grown in M9 minimal medium containing ^{15}N isotope for 17 hours at 21°C and after then 1 mM isopropyl-1-thio-β-D-thiogalactopyranoside was treated. His-tagged Dvl-1 was purified using Ni-NTA affinity chromatography, and imidazole, which was present at a high concentration, was removed using a PD-10 desalting column. After cleavage of the His-tag using the tobacco etch virus (TEV) protease, we conducted Ni-NTA affinity chromatogra`phy to obtain pure Dvl PDZ domain. Finally, Dvl PDZ was purified using size exclusion gel chromatography on a Superdex 75 column (Amersham Biosciences) equilibrated with 100 mM potassium phosphate pH 7.5, 0.5 mM EDTA and 2 mM DTT. The final concentration of Dvl-1 used in the nuclear magnetic resonance (NMR) titration was 0.6 mM.

KY-02061 and KY-02327 Synthesis

All solvents were dried by standard methods prior to use. Ethyl 5-benzyloxyindole-2-carboxylate and other chemicals were purchased from Aldrich Co. Analytical thin

layer chromatography (TLC) was performed on E. Merck pre-coated (0.25 mm) silica gel 60 F254 plates. Flash column chromatography was performed on silica gel 40 (Scientific Adsorbents Incorporated, Microns Flash). NMR experiments were performed using a deuterated solvent such as $CDCl_3$ as an internal standard.

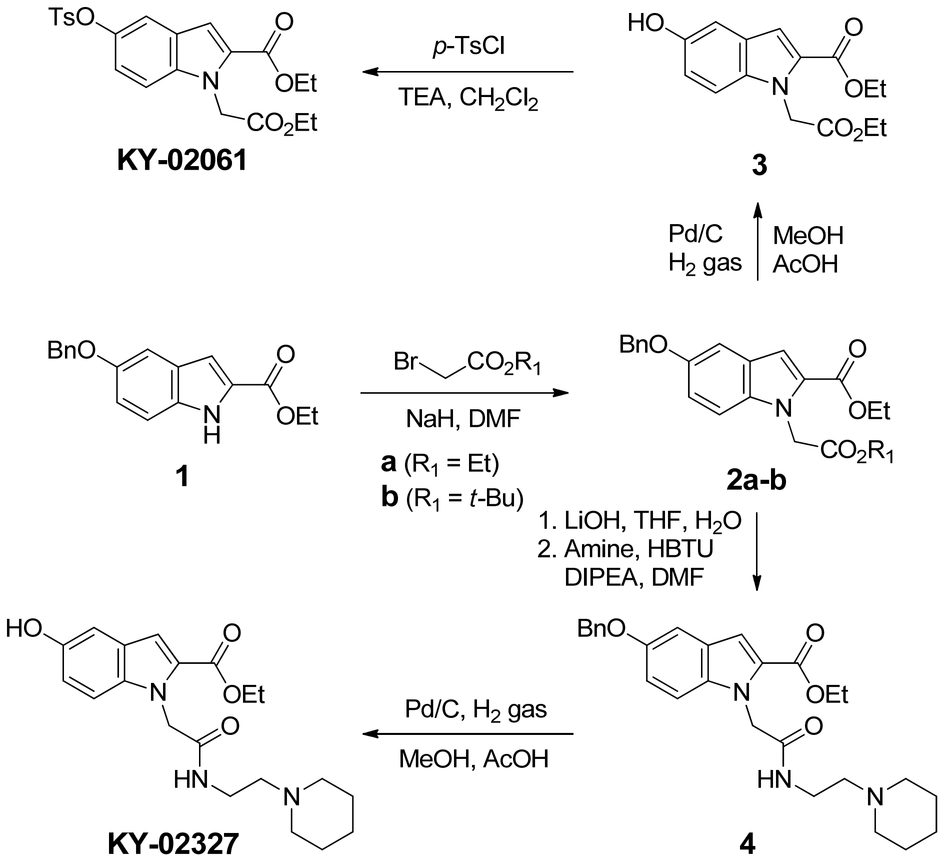

Appendix Scheme S1. KY-02061 and KY-02327 Synthesis

1. Ethyl 5-(benzyloxy)-1-(2-ethoxy-2-oxoethyl)-1H-indole-2-carboxylate synthesis. 5-Benzyloxyindole-2-carboxylate (**1**, 4.43 g, 15 mM) in DMF (50 ml) was added to NaH (60% dispersed in mineral oil, 0.9 g, 22.5 mM) portion-wise at 0°C. After 30 min stirring, ethyl bromoacetate (3.3 ml, 22.5 mM) was added drop-

wise. The resultant mixture was stirred at room temperature for 16 hours. After the reaction was completed, the excess NaH was quenched with water (50 ml), and the product was extracted with ethyl acetate (EtOAc, 50 ml, 3 times). The combined organic layer was dried over Na_2SO_4 and evaporated. The crude compound was purified by column chromatography to yield the title compound (**2a**) as a pale yellow solid (6.14 g, 99%). ^{1}H NMR (300 MHz, $CDCl_3$): δ 7.47-7.09 (m, 9H), 5.26 (s, 2H), 5.10 (s, 2H), 4.37-4.30 (q, J = 7.0 Hz, 2H), 4.23-4.17 (q, J = 7.0 Hz, 2H), 1.40-1.35 (t, J = 7.0 Hz, 3H), 1.27-1.22 (t, J = 7.0 Hz, 3H); ^{13}C NMR (76 MHz, DMSO): δ 169.1, 162.2, 154.2, 137.4, 135.2, 128.7, 128.1, 128.0, 127.7, 126.5, 117.6, 110.8, 110.7, 104.8, 70.8, 61.6, 60.8, 46.4, 14.4, 14.3; LC/MS(ESI): 382 ($M+H^+$).

2. *t*-butyl 5-(benzyloxy)-1-(2-ethoxy-2-oxoethyl)-1H-indole-2-carboxylate synthesis. The title compound (**2b**, 7.0 g, >99%) was prepared using similar reaction conditions to the synthesis of **2a**. ^{1}H NMR (300 MHz, $CDCl_3$): δ 7.50-7.32 (m, 5H), 7.28-7.11 (m, 3H), 5.20 (s, 2H), 5.12 (s, 2H), 4.40-4.32 (q, J = 6.0 Hz, 2H), 1.46 (s, 9H), 1.44-1.38 (t, J = 9.0 Hz, 3H); ^{13}C NMR (76 MHz, DMSO): δ 168.0, 162.0, 153.9, 137.3, 135.0, 128.5, 128.1, 127.9, 127.5, 126.3, 117.3, 110.6, 110.4, 104.5, 82.1, 70.6, 60.6, 46.9, 28.0, 14.3.; GC/MS(EI): 409 (M^+).

3. Ethyl 1-(2-ethoxy-2-oxoethyl)-5-hydroxy-1H-indole-2-carboxylate synthesis. The alkylated indole (**2a**, 0.3 g, 0.76 mM) was debenzylated by a Pd-catalyzed reaction using 10% Pd/C (30 mg) in MeOH (5 ml) and AcOH (0.1 ml) under balloon pressure of H_2 gas. After 4 hours stirring at room temperature, the mixture was filtered, and the solvent was evaporated. Then, the title compound (**3**) was purified by column chromatography (0.23 g, 99%). ^{1}H NMR ($CDCl_3$, 300 MHz): δ 7.23 (s, 1H), 7.15 (d, *J* = 8.9 Hz, 1H), 7.04 (s, 1H), 6.94 (d, *J* = 8.9 Hz, 1H), 5.26 (s, 2H), 4.34 (q, *J* = 7.1 Hz, 2H), 4.21 (q, *J* = 7.1 Hz, 2H), 1.38 (t, *J* = 7.1 Hz, 3H), 1.26 (t, *J* = 7.1 Hz, 3H); ^{13}C NMR (76 MHz, DMSO): δ 169.5, 162.3, 150.6, 135.1, 128.3, 126.8, 116.3, 110.5, 110.4, 106.4, 61.7, 60.9, 46.4, 14.4, 14.3; LC/MS(ESI): 292 ($M+H^+$).

4. Ethyl 1-(2-ethoxy-2-oxoethyl)-5-(tosyloxy)-1H-indole-2-carboxylate KY-02061 synthesis. The phenolic compound (**3**, 0.9 g, 3.1 mM) and TEA (0.47 ml, 3.4 mM) were dissolved in CH_2Cl_2 (10 ml), and then *p*-TsCl (0.65 g, 3.4 mM) was added. After 1 day stirring, the reaction mixture was diluted with water (10 ml), and the product was extracted with CH_2Cl_2 (10 ml, 3 times). The combined organic layer was dried over Na_2SO_4 and evaporated. The crude compound was purified by column chromatography to yield the title compound (**KY-02061**, 1.2 g, 85%). ^{1}H NMR (300 MHz, $CDCl_3$): δ 7.70 (d, *J* = 8.0 Hz, 2H), 7.30-7.27 (m, 3H), 7.17 (d, *J*

= 9.1 Hz, 1H), 7.00 (d, *J* = 9.1 Hz, 1H), 5.25 (s, 2H), 4.34 (q, *J* = 7.2 Hz, 2H), 4.21 (q, *J* = 7.1 Hz, 2H), 2.44 (s, 3H), 1.37 (t, *J* = 7.2 Hz, 3H), 1.26 (t, *J* = 7.1 Hz, 3H); ^{13}C NMR (76 MHz, DMSO): δ 168.7, 161.9, 145.3, 144.3, 137.7, 132.5, 129.8, 129.3, 128.7, 126.1, 120.5, 116.0, 111.3, 110.5, 61.8, 61.1, 46.5, 21.8, 14.4, 14.3; LC/MS(ESI): 446 ($M+H^+$).

5. Ethyl 5-(benzyloxy)-1-(2-oxo-2-((2-(piperidin-1-yl)ethyl)amino)ethyl)-1H-indole-2-carboxylate synthesis. The *N*-alkylated compound (**2b**, 6.1 g, 15 mM) in CH_2Cl_2 (150 ml) was treated with TFA (11.4 ml, 150 mM). The resultant mixture was stirred at room temperature for 10 hours. After evaporation, the hydrolyzed product (5.3 g, 99%) was obtained as a pale-yellow solid. The resultant acid (2.8 g, 8.0 mM), together with 2-aminoethyl-1-piperidine (3.1 g, 24 mM), HBTU (7.6 g, 20 mM), and DIPEA (3.1 g, 24 mM), was dissolved in DMF (27 ml) and stirred at room temperature overnight. Then, the reaction mixture was diluted with water (30 ml), and the product was extracted with EtOAc (30 ml, 3 times). The combined organic layer was dried over Na_2SO_4 and evaporated. The crude compound was purified by column chromatography to yield the title compound (**4**, 3.7 g, >99%). ^{1}H NMR (300 MHz, DMSO: δ 8.22 (br s, 1H), 7.45-7.02 (m, 9H), 5.14 (s, 2H), 5.09 (s, 2H), 4.27-4.20 (q, J = 6.0 Hz, 2H), 3.04-3.2.86 (m, 6H), 1.71-1.54 (m, 6H), 1.30-1.25 (t, J = 6.0 Hz, 3H); ^{13}C NMR (76 MHz, DMSO): δ 168.9, 161.7, 153.8,

137.7, 135.6, 128.8, 128.4, 128.0, 126.1, 122.6, 117.2, 112.3, 110.2, 105.7, 104.5, 70.0, 60.7, 55.3, 52.9, 34.3, 23.2, 21.6, 14.6; LC/MS(ESI): 464 ($M+H^+$).

6. Ethyl 5-hydroxy-1-(2-oxo-2-((2-(piperidin-1-yl)ethyl)amino)ethyl)-1H-indole-2-carboxylate KY-02327 synthesis. The ouabain (OBn) compound (**4**, 1.2 g, 2.5 mM) was debenzylated using the same Pd-catalyzed method described above (3). The reaction produced **KY-02327** (0.93 g, >99%) as an off-white solid after purification using column chromatography. ^{1}H NMR (300 MHz, CD_3OD): δ 7.32-7.29 (d, J = 9.0 Hz, 1H), 7.21 (s, 1H), 7.02-7.01 (d, J = 3.0 Hz, 1H), 7.01-6.92 (dd, J = 9.0, 3.0 Hz, 1H), 5.17 (s, 2H), 4.36-4.29 (q, J = 6.0 Hz, 2H), 3.59-3.55 (t, J = 6.0 Hz, 2H), 3.30-3.19 (m, 6H), 1.83-1.62 (m, 6H), 1.40-1.36 (t, J = 6.0 Hz, 3H); ^{13}C NMR (76 MHz, DMSO): δ 169.0, 161.8, 152.3, 134.9, 128.0, 126.6, 116.8, 111.8, 109.7, 105.3, 60.7, 55.4, 52.9, 47.6, 34.3, 23.2, 21.6, 14.5; LC/MS(ESI): 374 ($M+H^+$).

APPENDIX TABLES

Appendix Table S1. Activities and metabolic stability of KY-02061 and KY-02327

Name	Dvl-CXXC5 *in vitro* binding IC_{50} (μM)[a]	Relative ALP activity (%)[b]	Metabolic Stability (%)	
			Rat	Human
KY-02061	24	173	5.1 ± 2.4	25.2 ± 3
KY-02327	3.1	227	11.9 ± 1.49	33.5 ± 6.34

[a]The Dvl-CXXC5 *in vitro* binding assay was performed with 0.1, 1, 3, 10, 30 and 100 μM of each compound. The IC_{50} values were calculated using linear regression.

[b]MC3T3E1 cells were cultured in differentiation media with 10 μM of each compound for 4 days and ALP activity levels were quantified to monitor osteogenic differentiation of the cells.

Appendix Table S2. List of pathway-specific target genes

Pathway	Target gene	Primer sequence	Reference
Wnt	*Axin2*	Fwd: 5'-AGGTCCTGGCAACTCAGTAACAG-3'<br>Rev: 5'- CGCGAACGGCTGCTTATT-3'	Railo *et al*, 2009
	Fos-like antigen 1 (*Fosl1*)	Fwd: 5'- ACCTTGTGCCAAGCATCGA-3'<br>Rev: 5'- AATGAGGCTGCACCATCCA-3'	Germann *et al*, 2003
	WNT1 inducible signaling pathway protein 1 (*Wisp1*)	Fwd: 5'-TGAGCATTTGGAGAGCCAAGA-3'<br>Rev: 5'- AGAAGATTAAGCAAGCCATGTCAGT-3'	Railo *et al*, 2009
TGFβ	*Activating transcription factor 4* (*Atf4*)	Fwd: 5'-CGATGCTCTGTTTCGAATGGA-3'<br>Rev: 5'- CCAACGTGGTCAAGAGCTCAT-3'	Ranganathan *et al*, 2007
	Cyclin-dependent kinase inhibitor 1B (*Cdkn1b*)	Fwd: 5'-TCTTCGGCCCGGTCAAT-3'<br>Rev: 5'- CATATCCCGGCAGTGCTTCT-3'	Donovan *et al*, 2002
	Epithelial membrane protein 1 (*Emp1*)	Fwd: 5'-GGCTGCTGGGTCACACACT-3'<br>Rev: 5'- CCTGAACCACCATCAGAACTTG-3'	Ranganathan *et al*, 2007

NFκB	*BCL2-like 1* (*Bcl2l1*)	Fwd: 5'-CTGTGCGTGGAAAGCGTAGA-3'<br>Rev: 5'- CCAACTTGCAATCCGACTCA-3'	Pahl, 1999
	Tumor necrosis factor (*Tnf*)	Fwd: 5'-TCCAGGCGGTGCCTATGT-3'<br>Rev: 5'- GCCCCTGCCACAAGCA-3'	Pahl, 1999
JAK /STAT	*Suppressor of cytokine signaling 3* (*Socs3*)	Fwd: 5'-CGCGCACAGCCTTTCAG-3'<br>Rev: 5'- CCGGCCGGTCTTCTTGT-3'	Murray, 2007
p53	*BCL2-associated X protein* (*Bax*)	Fwd: 5'-GGCCTTTTTGCTACAGGGTTT-3'<br>Rev: 5'- GTGTCTCCCCAGCCATCCT-3'	Bouvard *et al*, 2000
	BCL2 binding component 3 (*Bbc3*)	Fwd: 5'-TGAACACTCTTTTTGTTAGCTTTACCA-3'<br>Rev: 5'- CACCGTTTCTTTCATGTCCTTACA	Han *et al*, 2001
Notch	*Hairy and enhancer of split 1* (*Hes1*)	Fwd: 5'-CCCCAGCCAGTGTCAACAC-3'<br>Rev: 5'- TGTGCTCAGAGGCCGTCTT-3'	Schwanbeck *et al*, 2011
	Jagged 1 (*Jag1*)	Fwd: 5'-ACACAGGGATTGCCCACTTC-3'<br>Rev: 5'- AGCCAAAGCCATAGTAGTGGTCAT-3'	Schwanbeck *et al*, 2011
PPAR	*Acyl-CoA synthetase long-chain family member 3* (*Acsl3*)	Fwd: 5'-CGCTGCACAGGCGTGTT-3'<br>Rev: 5'- ATGGCTGGACCTCCCAGAGT-3'	Cao *et al*, 2010
	Oxidized low density lipoprotein receptor 1 (*Olr1*)	Fwd: 5'-CCTGTTGCCGCATGAAAGA-3'<br>Rev: 5'- CCCTCTGCCTGCACTTGAG-3'	Chui *et al*, 2005
Oxidative stress	*Ferritin heavy polypeptide 1* (*Fth1*)	Fwd: 5'-AAGATGGGTGCCCCTGAAG-3'<br>Rev: 5'- CCAGGGTGTGCTTGTCAAAGA-3'	Huang *et al*, 2013
	Glutamate-cysteine ligase, catalytic subunit (*Gclc*)	Fwd: 5'-GGCCACTATCTGCCCAATTG-3'<br>Rev: 5'- CACGTAGCCTCGGTAAAATGG-3'	Han *et al*, 2008
Hypoxia	*Adrenomedullin* (*Adm*)	Fwd: 5'-GCCAGGCTCATGCCAGAA-3'<br>Rev: 5'- CCCCTGCCCGGAGAGTA-3'	Ke & Costa, 2006
	Erythropoietin (Epo)	Fwd: 5'-CCCCCACGCCTCATCTG-3'<br>Rev: 5'- TGCCTCCTTGGCCTCTAAGA-3'	Ke & Costa, 2006
	Vascular endothelial growth factor a (*VEGFa*)	Fwd: 5'-TCTTCAAGCCGTCCTGTGTG-3'<br>Rev: 5'-ATGTTGCTCTCTGACGTGGG-3'	Forsythe *et al*, 1996

Hedgehog	*Bone morphogenetic protein 2* (*BMP2*)	Fwd: 5'-ATCCAGTCTTGCCGCCTC-3'<br>Rev: 5'- GCCTCCTCCTCCTTCTCC-3'	Marigo *et al*, 1996
	Patched 1 (*Ptch1*)	Fwd: 5'-GCTGGAGGAGAACAAGCAAC-3'<br>Rev: 5'- GTCAAATGCATCCTGAAGTC-3'	Marigo *et al*, 1996
Control	β-actin	Fwd: 5'-GGATGCAGAAGGAGATTACT-3'<br>Rev: 5'- CCGATCCCACACAGAGTACTT-3'	-

Appendix Table S3. Bacterial reverse mutation assay with KY-02327

Tester Strain	Name	Dose (μg/plate)	Revertant colonies/pate (Mean) [Factor][a]	
			Without S-9 mix	With S-9 mix
TA-98	Vehicle[b]	0	19 ± 4	19 ± 2
	KY-02327	1000	19 ± 4 [1.0]	16 ± 1 [0.8]
	2-NF[c]	1	178 ± 16 [9.4]	n.a.
	B.P[c]	2	n.a.	205 ± 9 [10.8]
TA-100	Vehicleb	0	122 ± 4	117 ± 4
	KY-02327	1000	123 ± 6 [1.0]	117 ± 8 [1.0]
	S.A[c]	1	689 ± 20 [5.6]	n.a.
	B.P[c]	2	n.a.	724 ± 24 [6.2]

[a]Number of revertant colonies of treated plate/Number of revertant colonies of vehicle control plate (Maron & Ames, 1983).

[b]Negative control. Vehicle: DMSO

[c]Positive control. S.A: Sodium azide, 2-NF: 2-nitrofluorene, B.P: Benzo(a)pyrene,

Appendix Table S4. Cytochrome P450 (CYP) inhibition assay with 10 μM KY-02327

Compounds	Inhibition (%)		
	1A2	C29	3A4
KY-02327	0.0	0.0	0.0
a-Naphthoflavone	95.0	n.a.	n.a.
Sulfaphenazole	n.a.	80.5	n.a.
Ketoconazole	n.a.	n.a.	93.5

[a]Inhibition was measured using fluorogenic P450 substrates (Marks *et al*, 2002).

Appendix Table S5. Human Ether-à-go-go Related Gene (hERG) binding assay with 10 μM KY-0232

Compounds	Inhibition (%)[a]
KY-02327	10.87±2.069

[a]Inhibition was measured using fluorescence polarization method (Piper *et al*, 2008).

APPENDIX FIGURES AND FIGURE LEGENDS

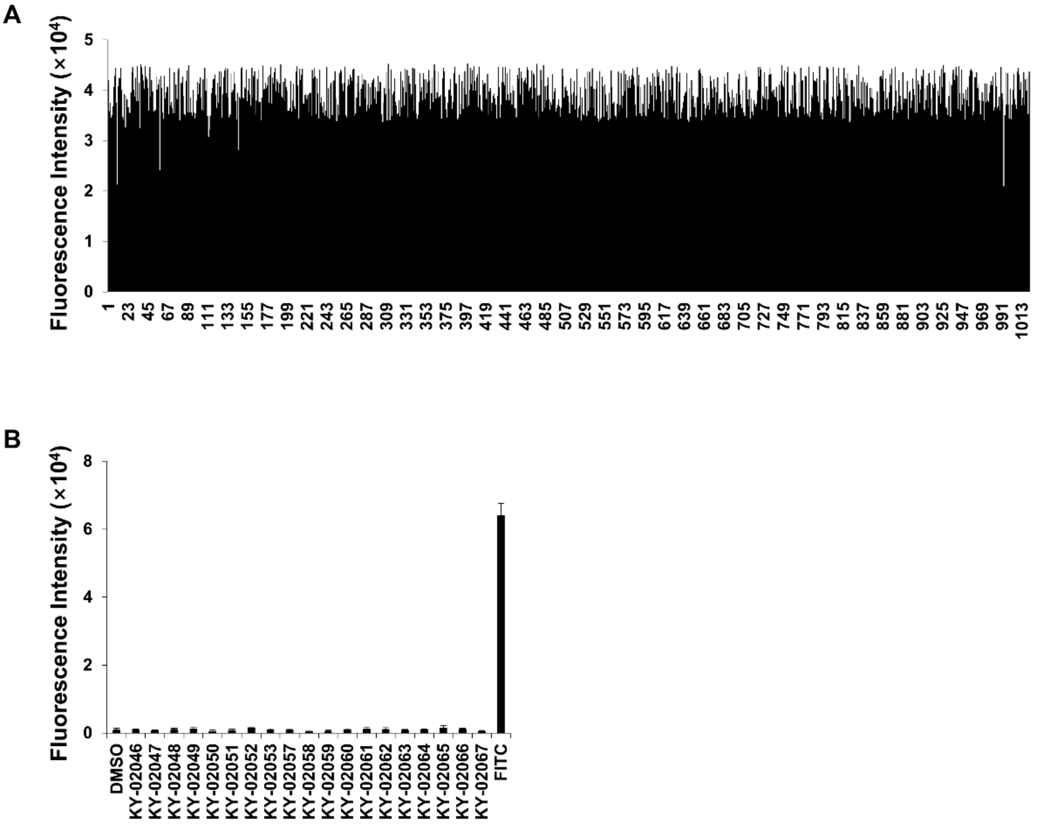

Appendix Figure S1. Screening and identification of DBM-mimetic small molecules.

A   A Dishevelled (Dvl)-CXXC5 *in vitro* binding assay using polyarginine conjugated Dvl binding motif (PolyR-DBM) was performed for 2 μM compounds from commercial library.

B   Autofluorescence of the compounds (each 2 μM) containing an indole ring and a carboxylic acid group was measured using a Fluorstar Optima microplate reader. The data are the mean ± s.d. (error bars). [*n*=2]

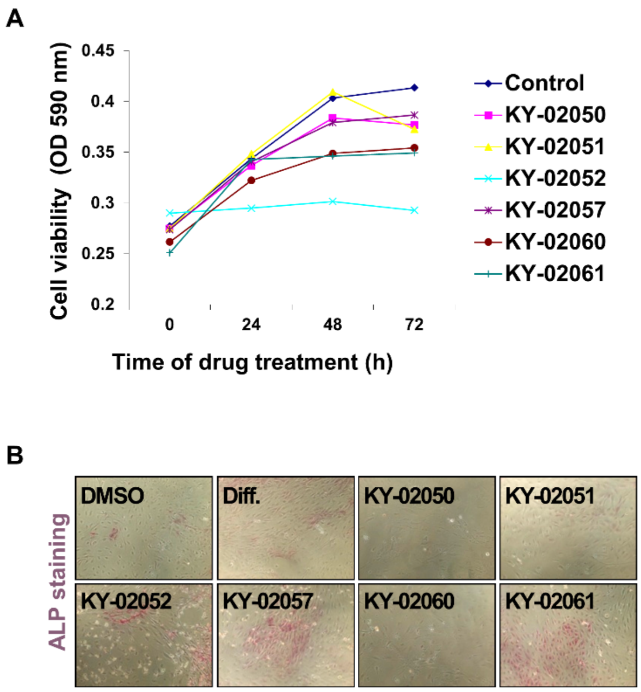

Appendix Figure S2. The effects of Dvl-CXXC5 binding small molecule inhibitors on primary calvaria cells.

A Primary calvaria cells were treated with 2 μM of each compound for the indicated times. The cells were subjected to an MTT assay (OD_{590} reading) to measure cell viability. The data are the mean ± s.d. (error bars). [*n*=3]

B Primary calvaria cells were treated with 2 μM of each compound for 4 days. ALP activity levels were visualized by ALP staining. [*n*=2]

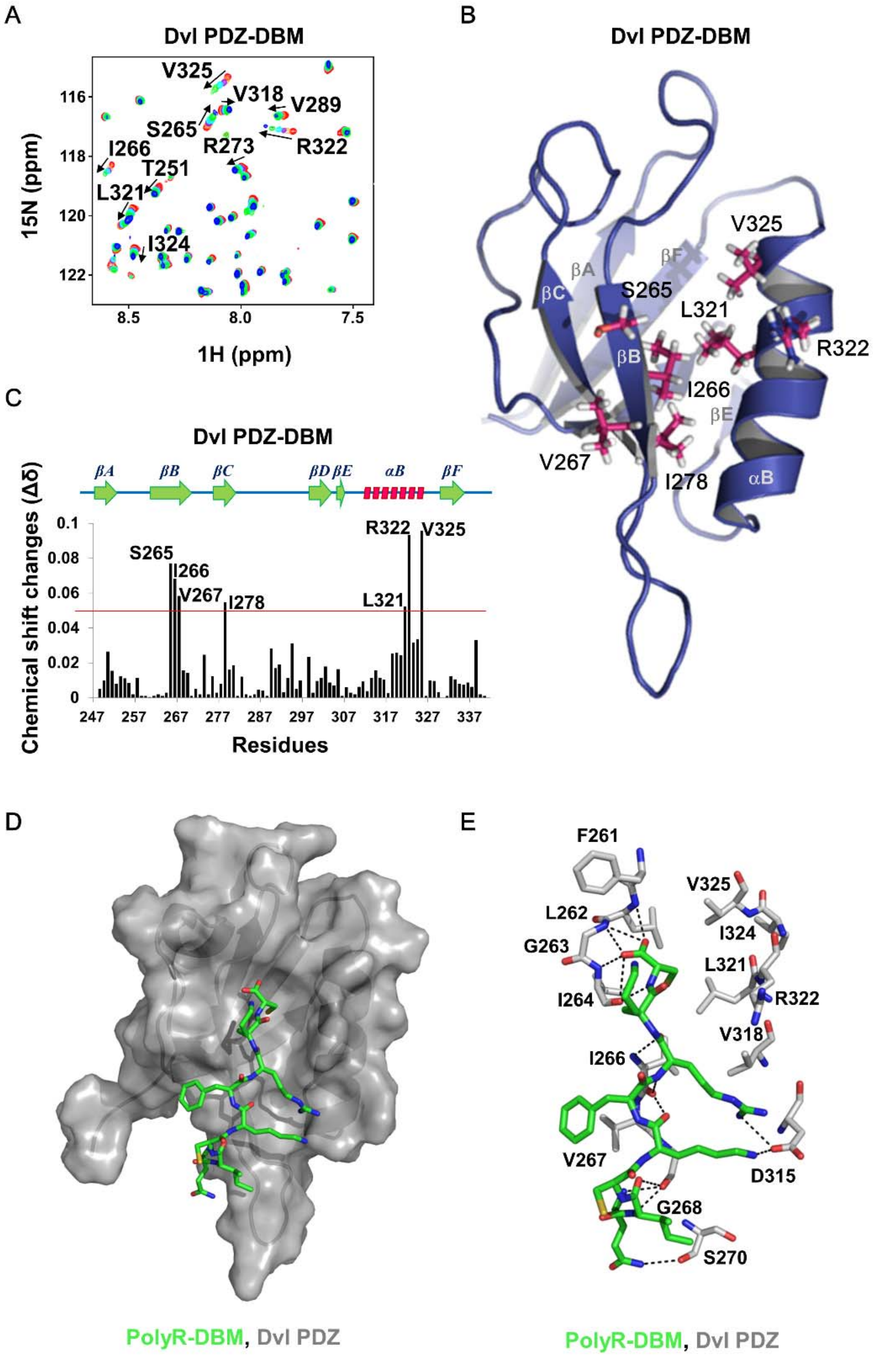

Appendix Figure S3. The binding of DBM to Dvl PDZ domain.

A-C NMR titration analyses for Dvl PZD domain with DBM. ^{1}H-^{15}N-HSQC analyses were performed to analyze the interaction of ^{15}N-labelled Dvl-PDZ domain with DBM. The ^{1}H-^{15}N-HSQC spectrum of different molar

ratios (Dvl PDZ domain:DBM) are displayed as red (1:0), orange (1:5), purple (1:10), cyan (1:15), green (1:20) and blue (1:25) (A, residues with meaningful chemical shift change are indicated by arrows). The residues with chemical shift changes ($\Delta\delta$) greater than 0.05 are visualized as a stick model on the ribbon representation of the Dvl PDZ domain structure (B). Plot of $\Delta\delta$ as a function of residue number in molecular ratio 1:25 (C, a red-colored line indicates the line for $\Delta\delta$=0.05).

D, E Molecular docking of DBM to Dvl PDZ domain were analyzed by *in silico* experiments. Docked binding mode of peptide DBM (stick representation) to the surface of Dvl PDZ domain (gray) (D). Detailed interactions between the Dvl PDZ domain and DBM (only the last six C-terminal amino acids [QICKFRKC] are shown). Key binding residues from the binding site are highlighted in gray and hydrogen bonds are represented by black as dotted lines (E).

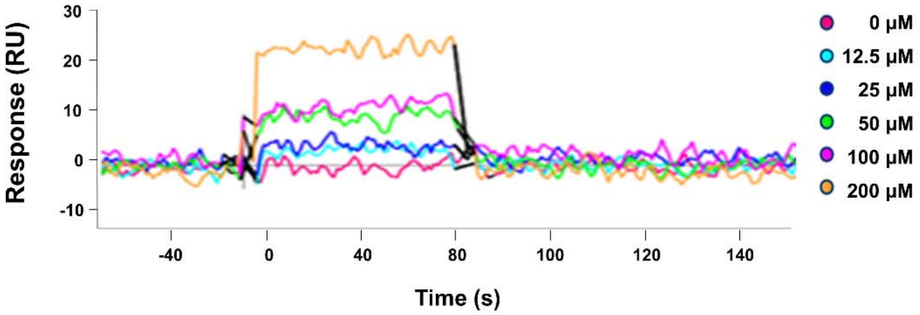

Appendix Figure S4. Surface plasmon resonance analysis for DBM-Dvl PDZ domain interaction.

KY-02327 dilutes of six different concentrations (0, 12.5, 25, 50, 100, and 200 μM) were flowed over a Dvl PDZ domain immobilized surface plasmon resonance sensor chip. The change of refractive index over the time in the surface of chip was measured and displayed as a graph.

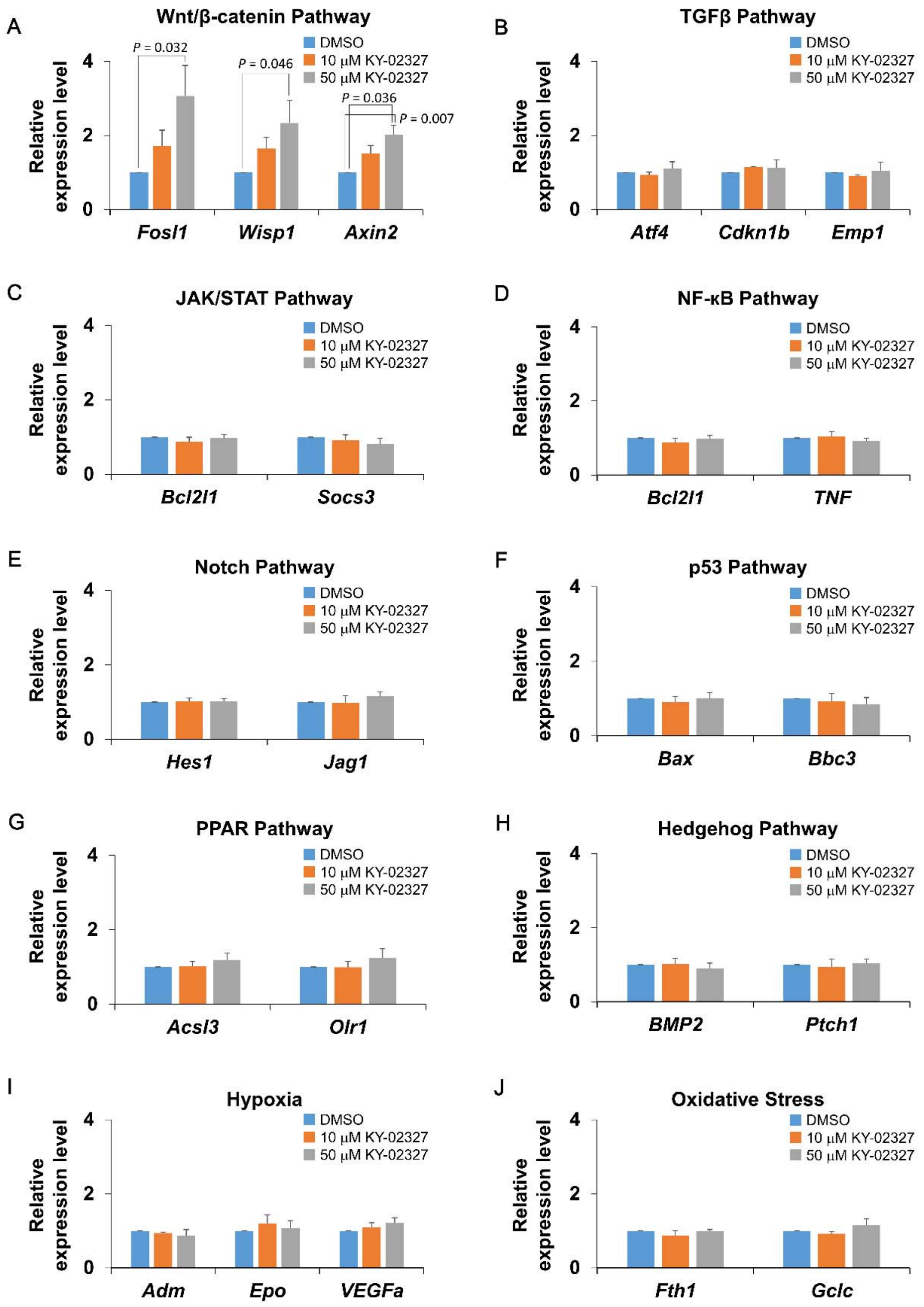

Appendix Figure S5. Quantitative real-time PCR analyses for target-specificity of KY-02327.

KY-02327 dilutes of three different concentrations (0, 10, and 50 μM) were treated

on mouse embryonic fibroblast cells for 12 hours. The mRNA level of pathway-specific target genes (Appendix Table S2) was measured by quantitative real-time PCR.

Data Information: The data are the mean ± s.d. (error bars), and significance was assessed using unpaired Student's t-test. [*n*=3]

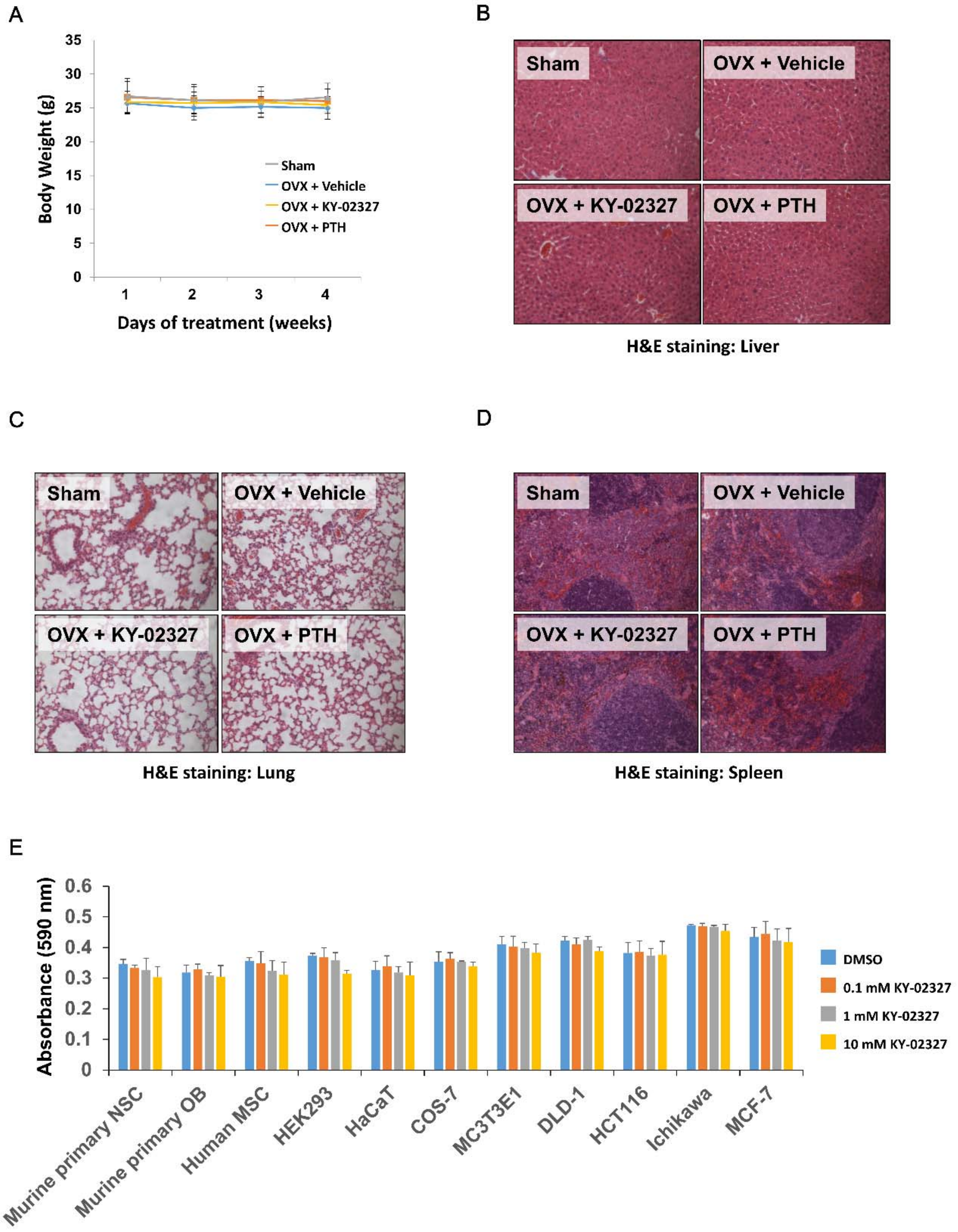

Appendix Figure S6. The toxicity test of KY-02327.

A-D Vehicle or 20 mg of KY-02327 per kg of animal body weight (mpk) was orally administered or 0.06 mpk of the N-terminal fragment of PTH

(amino acids 1-34) was subcutaneously injected into the Sham-operated (Sham) or ovariectomized (OVX) mice on 5 sequential days per week for 4 weeks [*n*=4]. During treatment, the weights of each mouse were measured and recorded every week (A). After 4 weeks treatment, mice were scarified and their organs were subjected to histological analyses. Hematoxylin and Eosin staining of liver (B), lung (C), and spleen (D).

E Various cells were treated with DMSO or 0.1, 1, or 10 μM KY-02327. After 48 hours, cells were subjected to an MTT assay (OD_{590} reading) to measure cell viability. NSC: neural stem cell, OB: osteoblast, MSC: mesenchymal stem cell. The data are the mean ± s.d. (error bars). [*n*=3]

APPENDIX REFERENCES

Bouvard V, Zaitchouk T, Vacher M, Duthu A, Canivet M, Choisy-Rossi C, Nieruchalski M, May E (2000) Tissue and cell-specific expression of the p53-target genes: bax, fas, mdm2 and waf1/p21, before and following ionising irradiation in mice. *Oncogene* 19**:** 649-660

Cao A, Li H, Zhou Y, Wu M, Liu J (2010) Long chain acyl-CoA synthetase-3 is a molecular target for peroxisome proliferator-activated receptor delta in HepG2 hepatoma cells. *J Biol Chem* 285**:** 16664-16674

Chui PC, Guan H-P, Lehrke M, Lazar MA (2005) PPARgamma regulates adipocyte cholesterol metabolism via oxidized LDL receptor 1. *J Clin Invest* 115**:** 2244-2256

Donovan JCH, Rothenstein JM, Slingerland JM (2002) Non-malignant and tumor-derived cells differ in their requirement for p27Kip1 in transforming growth factor-beta-mediated G1 arrest. *J Biol Chem* 277**:** 41686-41692

Forsythe JA, Jiang BH, Iyer NV, Agani F, Leung SW, Koos RD, Semenza GL (1996) Activation of vascular endothelial growth factor gene transcription by hypoxia-inducible factor 1. *Mol Cell Biol* 16**:** 4604-4613

Germann A, Dihlmann S, Hergenhahn M, Doeberitz MvK, Koesters R (2003) Expression profiling of CC531 colon carcinoma cells reveals similar regulation of beta-catenin target genes by both butyrate and aspirin. *Int J Cancer* 106**:** 187-197

Han E-S, Muller FL, Pérez VI, Qi W, Liang H, Xi L, Fu C, Doyle E, Hickey M, Cornell J, Epstein CJ, Roberts LJ, Van Remmen H, Richardson A (2008) The in vivo gene expression signature of oxidative stress. *Physiol Genomics* 34**:** 112-126

Han J, Flemington C, Houghton AB, Gu Z, Zambetti GP, Lutz RJ, Zhu L, Chittenden T (2001) Expression of bbc3, a pro-apoptotic BH3-only gene, is regulated by diverse

cell death and survival signals. *Proc Natl Acad Sci U S A* 98**:** 11318-11323

Huang B-W, Ray PD, Iwasaki K, Tsuji Y (2013) Transcriptional regulation of the human ferritin gene by coordinated regulation of Nrf2 and protein arginine methyltransferases PRMT1 and PRMT4. *FASEB J* 27**:** 3763-3774

Ke Q, Costa M (2006) Hypoxia-inducible factor-1 (HIF-1). *Mol Pharmacol* 70**:** 1469-1480

Marigo V, Scott MP, Johnson RL, Goodrich LV, Tabin CJ (1996) Conservation in hedgehog signaling: induction of a chicken patched homolog by Sonic hedgehog in the developing limb. *Development* 122**:** 1225-1233

Marks BD, Smith RW, Braun HA, Goossens TA, Christenson M, Ozers MS, Lebakken CS, Trubetskoy OV (2002) A high throughput screening assay to screen for CYP2E1 metabolism and inhibition using a fluorogenic vivid p450 substrate. *Assay Drug Dev Technol* 1**:** 73-81

Maron DM, Ames BN (1983) Revised methods for the Salmonella mutagenicity test. *Mutat Res* 113**:** 173-215

Murray PJ (2007) The JAK-STAT signaling pathway: input and output integration. *J Immunol* 178**:** 2623-2629

Pahl HL (1999) Activators and target genes of Rel/NF-kappaB transcription factors. *Oncogene* 18**:** 6853-6866

Piper DR, Duff SR, Eliason HC, Frazee WJ, Frey EA, Fuerstenau-Sharp M, Jachec C, Marks BD, Pollok BA, Shekhani MS, Thompson DV, Whitney P, Vogel KW, Hess SD (2008) Development of the predictor HERG fluorescence polarization assay using a membrane protein enrichment approach. *Assay Drug Dev Technol* 6**:** 213-223

Railo A, Pajunen A, Itäranta P, Naillat F, Vuoristo J, Kilpeläinen P, Vainio S (2009) Genomic response to Wnt signalling is highly context-dependent--evidence from DNA microarray and chromatin immunoprecipitation screens of Wnt/TCF targets. *Exp Cell*

Res 315**:** 2690-2704

Ranganathan P, Agrawal A, Bhushan R, Chavalmane AK, Kalathur RKR, Takahashi T, Kondaiah P (2007) Expression profiling of genes regulated by TGF-beta: differential regulation in normal and tumour cells. *BMC Genomics* 8**:** 98

Schwanbeck R, Martini S, Bernoth K, Just U (2011) The Notch signaling pathway: molecular basis of cell context dependency. *Eur J Cell Biol* 90**:** 572-581
